# Clinical characteristics and outcomes among patients admitted with viral hemorrhagic fever at Mbarara Regional Referral Hospital, Uganda

**DOI:** 10.1016/j.ijregi.2026.101030

**Published:** 2026-10-11

**Authors:** Rose Muhindo, Ernest Ssewanyana, Badru Kayongo, Andrew Mutekanga, Francis Mugabi, Precious Twesigye, Jeremiah Lwasa, Nelson Wandera, Peter K. Olds, Louise C. Ivers

**Affiliations:** 1 Department of Internal Medicine, Mbarara University of Science and Technology, Mbarara, Uganda; 2 School of Public Health, Imperial College London, London, United Kingdom; 3 Department of Psychiatry, Mbarara Regional Referral Hospital, Mbarara, Uganda, Uganda; 4 Department of Community Health, Mbarara Regional Referral Hospital, Mbarara, Uganda; 5 Department of Medicine, Divine Mercy Hospital, Mbarara, Uganda; 6 Department of Medicine, Mbarara Regional Referral Hospital, Mbarara, Uganda; 7 Centre for Global Health, Massachusetts General Hospital, Boston, MA, USA; 8 Department of Global Health and Social Medicine, Harvard Medical School, Boston, MA, USA

**Keywords:** Viral hemorrhagic fever, Rift Valley fever, Crimean-Congo hemorrhagic fever, Uganda, Clinical characteristics

## Abstract

**Objectives:**

Viral hemorrhagic fevers (VHFs), such as Rift Valley fever and Crimean-Congo hemorrhagic fever, pose serious public health concerns in endemic regions, such as Uganda, where outbreaks occur sporadically. Despite this, there are limited data on the clinical characteristics and outcomes of affected patients in rural settings.

**Methods:**

This is a retrospective descriptive study of 36 patients with laboratory-confirmed VHF who were treated at the isolation unit of Mbarara Regional Referral Hospital in southwestern Uganda during the outbreaks that occurred between 2018 and 2024. Data on demographics, clinical presentation including laboratory parameters, complications, and outcomes were extracted from the patients’ medical records and analyzed using descriptive statistics. The Welch’s *t*-test was used to describe the difference in length of hospital stay between the survivors and patients who died of VHF.

**Results:**

Of the 36 patients included, 88.9% (n = 32) were male, with a median age of 35 (interquartile range [IQR] 28-42.5) years. A total of 69.4% (n = 25) of the patients interacted directly with animals or animal products in their occupations. The most common presenting symptoms included bleeding tendencies (28 [77.8%]), fever (23 [63.9%]), and vomiting (19 [52.8%]). The median duration of symptoms was 5 days (IQR 4-7). Laboratory results revealed severe kidney impairment, with a median creatinine of 10.3 mg/dL (IQR 7.46-13), elevated aspartate aminotransferase of 1014 (IQR 173-2570) IU/l and alanine aminotransferase of 795.9 (IQR 233-1634) IU/l, prolonged international normalized ratio (median 1.65 [IQR 1.5-1.7]), hyperbilirubinemia (mean serum bilirubin 5.7 [SD 1.2]), and thrombocytopenia (median platelet count 43,000 [IQR 16,000-65,000]). The overall case fatality rate was 41.7% (n = 15), with a mean time from admission to death of 2.40 [SD 2.75] days. There was a statistically significant difference in the mean length of hospital stay between the survivors and those who died in the hospital (mean difference 15.07 days; 95% confidence interval 11.57-18.57; *P* < 0.001).

**Conclusion:**

In southwestern Uganda, patients with VHFs present with severe illness characterized by hemorrhagic, hepatic, and renal complications. The high case fatality rate indicates the need for early detection and comprehensive supportive care interventions, such as hemodialysis.

## Highlights

• This study describes the presentation, management, and outcomes of viral hemorrhagic fever at a tertiary hospital.
• A total of 88.9% were men, and 69.4% interacted directly with animals in their occupations.
• Bleeding, fever, and vomiting were the most frequent symptoms.
• Most patients were managed supportively and 52.8% survived, whereas 41.7% died.
• Patients who died did so very early during admission, with a mean of 2.45 days.

## Background

Rift Valley fever (RVF) virus (RVFV) and Crimean-Congo hemorrhagic fever (CCHF) virus are among the nine World Health Organization priority pathogens with the potential to cause pandemics [1]. Although they primarily cause disease outbreaks in livestock and wild animals, they also periodically spill over into human populations, causing severe hemorrhagic fever outbreaks. There is particular concern about the growing incidence of RVF and CCHF outbreaks, making them re-emerging infectious diseases of public health importance [2,3]. Uganda is one of the countries that continues to experience these sporadic viral hemorrhagic fever (VHF) outbreaks. Other regions that experience RVF and CCHF outbreaks include the East African Rift Valley basin, tropical Africa, the Middle East, Eastern Europe, and Asia [4,5]. The most recent outbreaks of VHF in southwestern Uganda have included the RVF outbreaks of 2018, 2023, and 2024 and the CCHF outbreaks of 2018 and 2019 [6].

In Uganda, the first serological evidence of RVFV was reported in 1944 in mosquitoes of the Semliki rainforest in western Uganda [7]. Since then, outbreaks of RVF have continued to occur sporadically in the country (Figure 1), especially in the cattle-rearing regions of the cattle corridor (Figure 2), making Mbarara Regional Referral Hospital (MRRH) a key player in the management of RVF outbreaks [8,9]. Nonetheless, data on the clinical characteristics, management, and outcomes of RVF in Uganda are limited. Many patients with severe RVF present with bleeding from body orifices. Severe illness may also present with rapid deterioration of kidney function, meningoencephalitis, ocular complications, and liver failure [10]. Some patients, however, are asymptomatic or have nonspecific symptoms [11].

**Figure 1:**
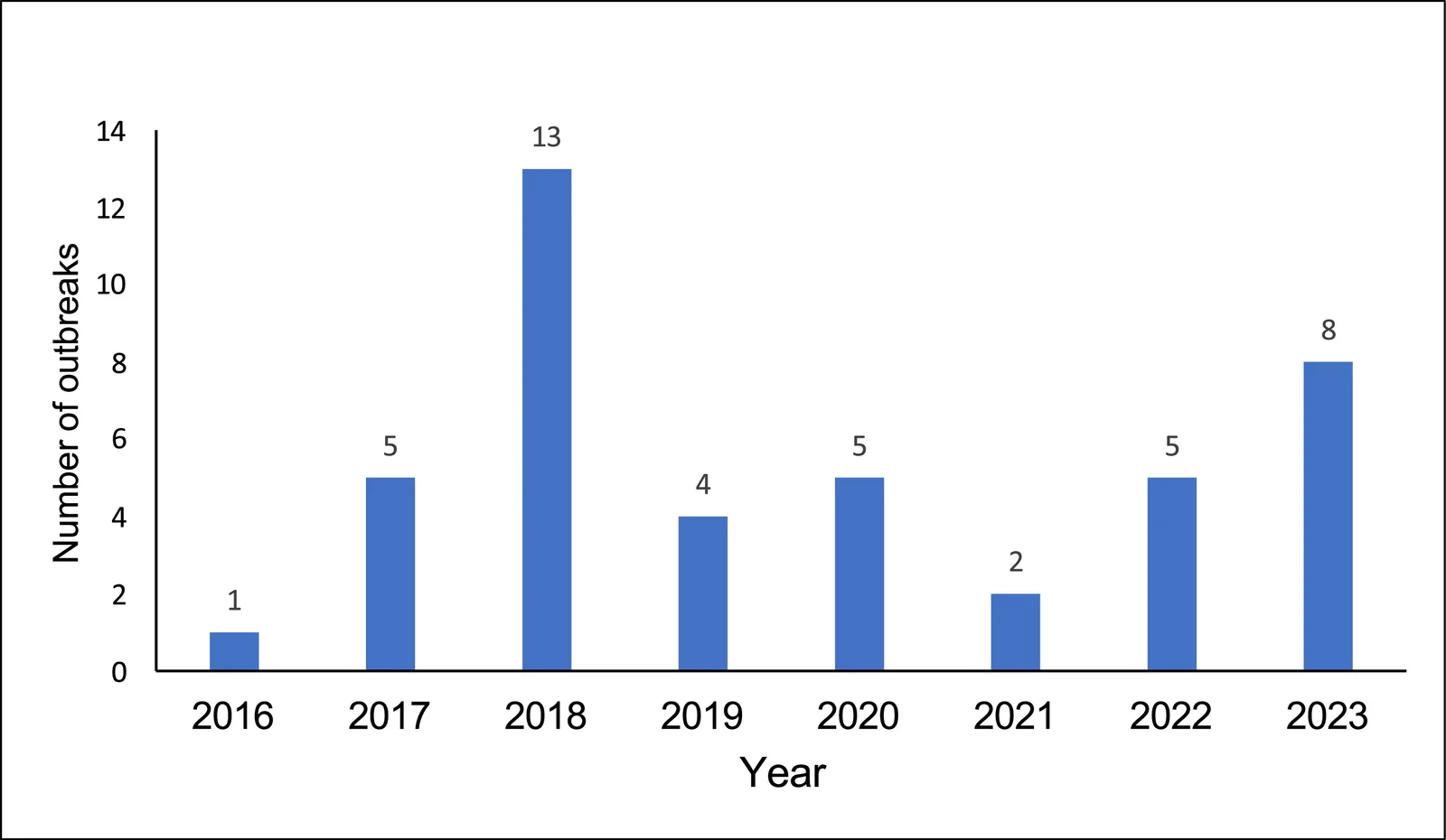
Rift Valley Fever Outbreaks in Uganda by year since 2016 [12]. Adapted from the Uganda National Institutes of Public Health, 2024.

**Figure 2:**
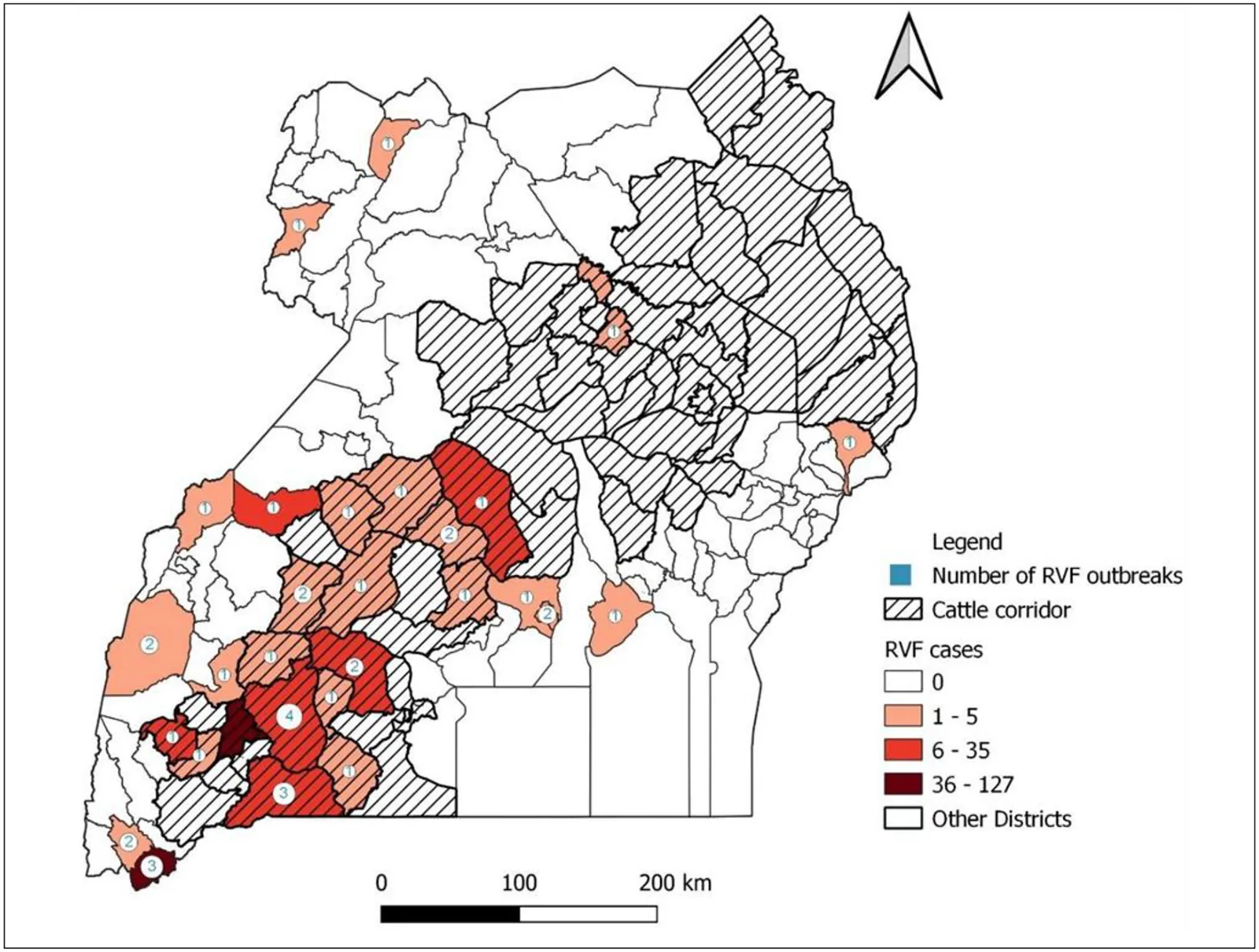
A map of Uganda showing the cattle corridor and number of RVF cases by district from 2016 to 2023 [12]. Adapted from the Uganda National Institutes of Public Health, 2024. Abbreviation: RVF, Rift Valley fever.

On the other hand, CCHF is a tick-borne viral disease caused by *Nairovirus* of the *Bunyaviridae* family. CCHF is classified as hazard group 4 by the Advisory Committee on Dangerous Pathogens, as are Ebola and Marburg, because of its ability to cause severe human disease with a high risk of spread and the absence of a known effective treatment or prophylaxis [13]. It causes epidemics in Africa, the Middle East, Eastern Europe, and Asia. Many areas in the Ugandan cattle corridor were afflicted by CCHF during the 2018 and 2019 outbreaks (Figure 3).

**Figure 3:**
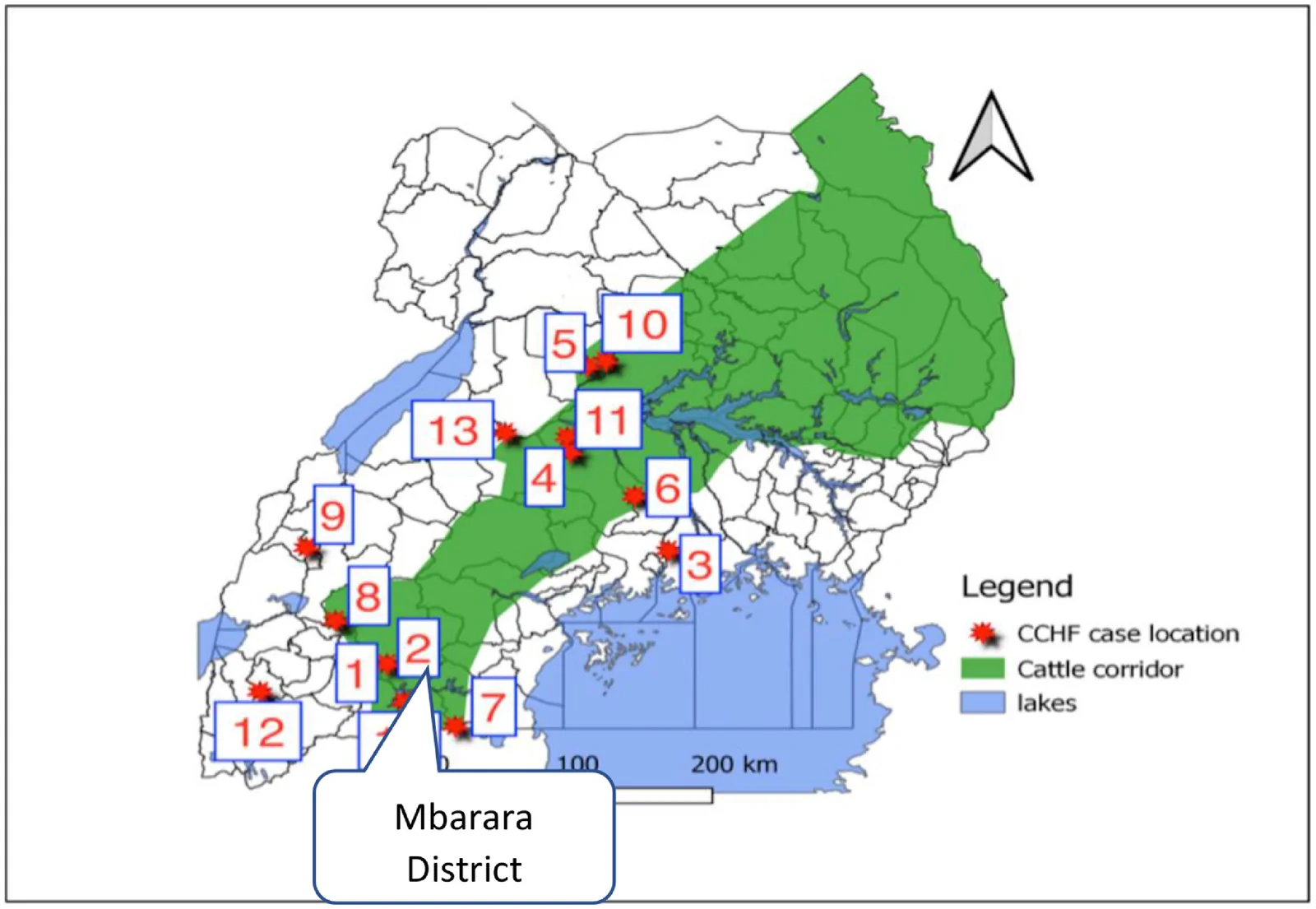
A map of Uganda showing the distribution of cases of CCHF by district in the 2018-2019 outbreak [6]. Adapted from Mirembe et al. [6]. Abbreviation: CCHF, Crimean-Congo hemorrhagic fever.

Although there are no proven anti-viral treatments for either RVF or CCHF, supportive symptomatic management is recommended and has been effective in many parts of the world [13]. Hemodialysis among patients with VHF and severe acute kidney injury has also improved outcomes in these patients, even at MRRH [14]. However, some patients present with severe forms of the disease and die. Therefore, the management of patients during an outbreak is significantly complicated by the broad range of acute and delayed symptoms with potentially negative outcomes [15,16]. As such, the case fatality rate in RVF has been reported to range from 0.3% to 44.7% [17]. Similarly, previous studies have reported case fatality rates for CCHF as high as 10%-40% [18].

This study presents a descriptive account of the clinical presentation, management, and in-hospital outcomes of patients with VHF at a tertiary government hospital in southwestern Uganda, East Africa, during three outbreaks of RVF between 2018 and 2024 and two outbreaks of CCHF in 2018 and 2019 (Figure 1).

### Objectives

The primary objective of this study was to describe the clinical presentation, management, and in-hospital outcomes of people diagnosed with RVF and CCHF at MRRH between 2018 and 2024.

## Methods

The authors used a retrospective study design by abstracting data from the medical records of patients admitted to MRRH with confirmed laboratory diagnoses of either RVF or CCHF from 2018 through 2024. A confirmed case was one with laboratory confirmation of the presence of RVF or CCHF ribonucleic acid by reverse transcription–polymerase chain reaction (PCR) [8]. A data extraction tool with sections on demographics, clinical presentation, management, and outcomes, including complications, was used to collect data from the medical records. Recovery was defined as the complete remission of symptoms with a negative ribonucleic acid PCR test for either RVFV or CCHF virus.

### Study site

MRRH in Mbarara City in southwestern Uganda was the study site. The hospital has a bed capacity of about 600 and serves at least 16 districts in the region, in addition to patients from neighboring countries, including Burundi, Democratic Republic of the Congo, Tanzania, and Rwanda. MRRH is the teaching hospital for Mbarara University of Science and Technology, Bishop Stuart University, and Mayanja Memorial Training Institute.

The hospital has a 40-bed capacity isolation unit/special pathogens unit, commissioned in 2021, which serves as a regional unit for managing people with VHF and other highly infectious diseases, including coronavirus disease 2019 and mpox. The unit is manned by physicians, medical officers, and nurses. The hospital also has a laboratory that can conduct hematology, chemistry, and microbiological tests. The confirmation of VHF infection, however, is done by reverse transcription–quantitative PCR test at the Uganda Virus Research Institute in Entebbe, about 292 km from Mbarara. Blood samples are drawn, packaged safely in biohazard bags, and transported by car to Uganda Virus Research Institute. Currently, the results are relayed through an electronic results dispatch system with an average turnaround time of 36-48 hours.

### Population

The study included the medical records of all admitted patients who had a laboratory-confirmed diagnosis of VHF at MRRH between January 2018 and December 2024.

### Study variables

The study variables included demographic information (age, gender, residence, and occupation), clinical presentations (symptoms and their duration), laboratory results including complete blood count, liver enzymes (alanine aminotransferase [ALT] and aspartate aminotransferase [AST]), kidney function tests (creatinine, urea, potassium, and sodium) and serological tests for RVF, treatment modalities, comorbidities, and outcomes (recovery, complications, and mortality). Acute kidney injury (AKI) was defined as a rise in serum creatinine by 1.5 times the baseline, which was known or presumed to have occurred within the past 7 days [12]. Acute liver injury (ALI) was defined as the presence of markers of liver damage, such as raised ALT and/or AST levels, jaundice, and coagulopathy (international normalized ratio [INR] >1.5) without encephalopathy in a person who did not have pre-existing liver disease [19]. Electrolyte imbalance was defined as an increase or decrease in the serum levels of sodium, potassium, and chloride ions above or below the reference ranges provided by the laboratory [20]. Anemia was defined as a measured hemoglobin level below 13 g/dL for men and below 12 g/dL for women [21].

### Data analysis

Data were summarized in Microsoft Excel before being exported to STATA 13 (StataCorp. (2013). Stata: Release 13. Statistical Software. College Station, TX: StataCorp LP) for analysis. Descriptive statistics were used to characterize the demographics, clinical features, and outcomes of patients with VHF. Frequencies and percentages were used to describe categorical variables. Means and SDs were used to describe continuous variables that were normally distributed. Medians and interquartile ranges were used to describe skewed data. Welch’s *t*-test was used to assess the difference in length of hospital stay between the survivors and patients who died of VHF.

### Ethical considerations

Ethical approval, including a waiver of consent, was obtained from the Mbarara University Research and Ethics Committee (MUST-2024-1552). Administrative clearance and permission to access the hospital records were obtained from the MRRH administration. Data were de-identified to maintain participant anonymity and confidentiality.

## Results

Among the 36 patients admitted to the isolation unit with confirmed VHF, 31 (86.1%) had RVF, and only five (13.9%) were diagnosed with CCHF (Table 1). A strong male predominance was observed, with 88.9% (n = 32) of cases occurring in men. The median age was 35 years (interquartile range [IQR] 28-42.5). A total of 80.6% (n = 29) of the study participants resided in rural areas. A total of 69.4% (n = 25) of the patients interacted directly with animals or animal products in their occupations. These included six butchers, four cattle keepers, one cultivator, one dairy operator, four farmers, five herdsmen, one meat roaster, and three peasants. Direct contact with animals could not be determined in 30.6% (n = 11) of the patients, of whom four were unemployed, one was a trader, one was a housewife, one was a builder, and one was a teacher. Three of the people who presented with CCHF were from a refugee camp, including two refugees and one assistant refugee settlement manager.

**Table 1:** Sociodemographic characteristics of the study participants.

Variable	n. (%)
Age, median (IQR), years	35 (28-42.5)
Males, n (%)	32 (88.9)
Occupation, n (%)	
Butcher	6 (16.7)
Cattle keeper	4 (11.1)
Herdsman	5(13.9)
Peasant	3(8.3)
Dairy operator	1(2.8)
Meat roaster	1 (2.8)
Residence, n (%)	
Rural	29 (80.6)
Urban	7 (19.4)
VHF category	
Rift Valley fever, n (%)	31 (86.1)
CCHF	5 (13.9)

Abbreviations: CCHF, Crimean-Congo hemorrhagic fever; IQR, interquartile range; VHF, viral hemorrhagic fever.

### Clinical characteristics

Bleeding was the most frequent presenting symptom, reported in 28 (77.8%) patients, and ranged from epistaxis and gum bleeding to gastrointestinal hemorrhage and hematuria (Table 2). Fever was present in 23 (63.9%) patients. Vomiting (19 [52.8%]), malaise (eight [22.2%]), and yellowing of the eyes (seven [19.4%]) were also common signs and symptoms. Altered mental status (five [13.9%]) and reduced urine output (four [11.1%]) were observed among patients with severe illness. The median duration of symptoms before hospitalization was 5 (IQR 4-7) days. Most patients with VHF had normal vital signs and were fully conscious on admission, with a mean systolic blood pressure of 120 (SD 18) mmHg, a median oxygen saturation of 97% (IQR 96%-98%), and a mean temperature of 36.7° (SD 0.9) (Table 2). The median Glasgow coma scale score was 15 (IQR 14-15).

**Table 2:** Clinical presentation (N = 36).

Clinical characteristics	n (%)
Bleeding	28 (77.8)
Fever	23 (63.9)
Vomiting	19 (52.8)
Malaise	8 (22.2)
Yellowing of eyes	7 (19.4)
Reduced level of consciousness/altered mental state	5 (13.9)
Reduced urine output	4 (11.1)
Diarrhea	2 (5.6)
Abdominal pain	5 (13.9)
Headache	10 (27.8)
Others	6 (16.7)
Duration of symptoms, median (IQR) days	5 (4-7)
**Vital signs**	
Temperature (°C), mean (SD)	36.7 (0.9)
Pulse rate, beats/min, mean (SD)	89 (15)
Systolic blood pressure, mmHg, mean (SD)	120 (18)
Oxygen saturation, %, median (IQR)	97 (96-98)
Glasgow coma scale, median (IQR)	15 (14-15)
**Laboratory findings**	
WBC × 10^3^/l, median, (IQR)	3.45(2.37-12.54)
Hemoglobin g/dL, mean (SD)	9.5 (3.2)
Platelets, median (IQR)	43000 (16000-65000)
Serum creatinine, mg/dL, median (IQR)	10.3 (7.46-13)
Urea, mg/dL, median (IQR)	191.1 (159.5-291.7)
Serum sodium, mmol/l, mean (SD)	127.4 (7.7)
Serum potassium, mmol/l, mean (SD)	4.7 (1.2)
Total bilirubin, mg/dL, mean (SD)	5.7 (1.2)
INR, median (IQR)	1.65 (1.5-1.7)
AST IU/l, median (IQR)	1014 (173-2570)
ALT IU/l, median (IQR)	795.9 (233-1634)

Abbreviations: ALT, alanine aminotransferase; AST, aspartate aminotransferase; INR, international normalized ratio; IQR, interquartile range; WBC, white blood cell.

### Laboratory findings

The mean hemoglobin in this study population was 9.5 g/dL (SD 3.2), and the median platelet count was 43,000/µL (IQR 16,000-65,000) (Table 2). AKI was also a common finding, with a median creatinine level of 10.3 mg/dL (IQR 7.46-13) and a median urea level of 191.1 mg/dL (IQR 159.5-291.7). Stage 3 AKI developed in 20 (55.6%) patients. Many patients also had ALI (Figure 6) with marked elevation of liver enzymes (median AST 1014 [IQR 173-2570] IU/l, ALT 795.9 [IQR 233-1634] IU/l) and coagulopathy (median INR 1.65 [IQR 1.5-1.7]).

### Treatment

The treatment offered to the study population was mostly supportive (Figure 4). This included antibiotics (28 [77.6%]) and analgesics (17 [47.2%]). Tranexamic acid was administered to 13 (36.1%) patients, whereas three (8.3%) received intravenous vitamin K supplementation. Of the 20 patients who developed stage 3 AKI and therefore required hemodialysis, only 12 (60%) received the intervention. This was because hemodialysis is a paid-for service at the study center, and it was unaffordable to those who did not receive it.

**Figure 4:**
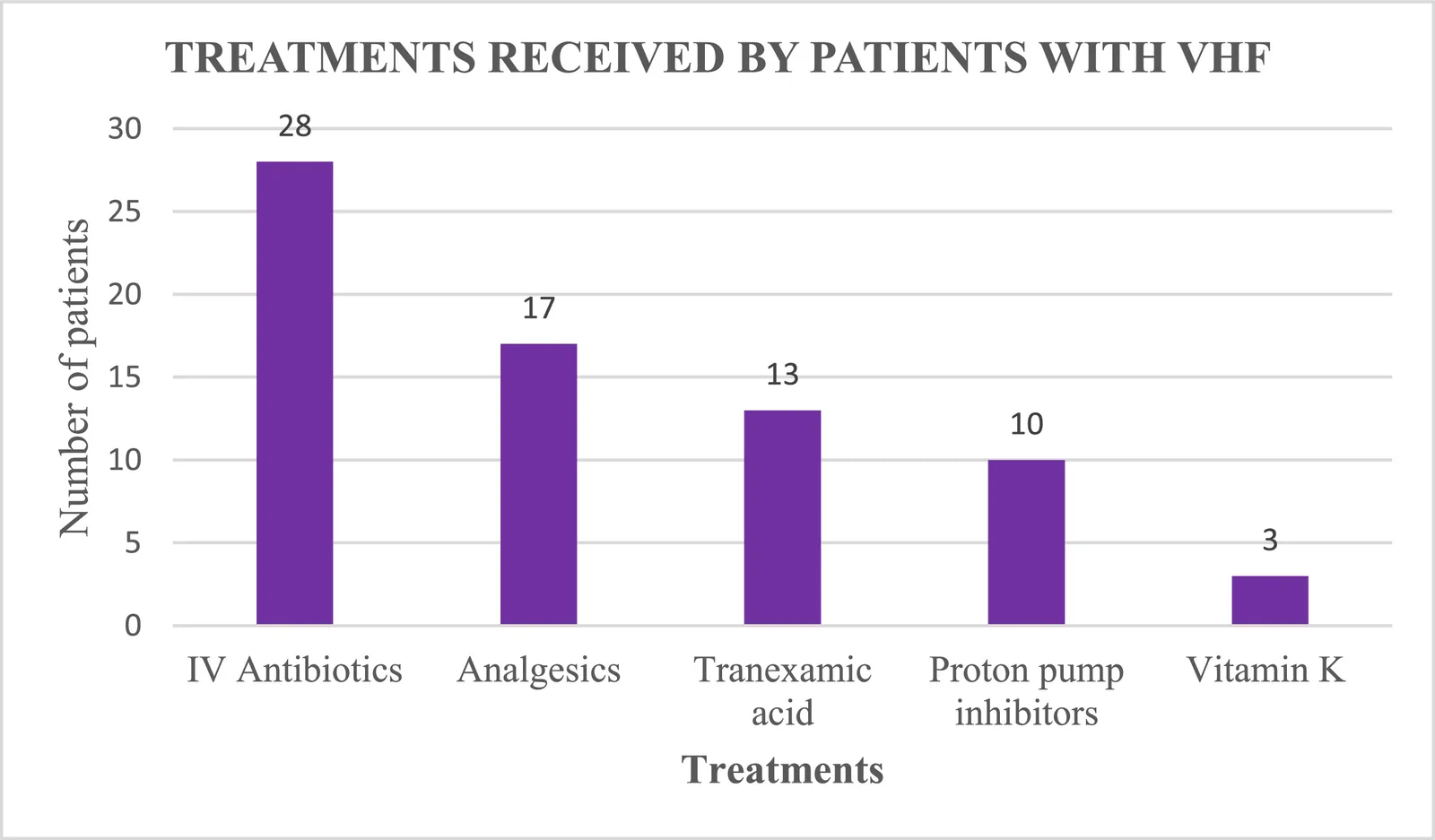
Treatments received by patients with RVF and CCHF at MRRH, southwestern Uganda. Abbreviations: RVF, Rift Valley fever; CCHF, Crimean-Congo hemorrhagic fever; MRRH, Mbarara Regional Referral Hospital.

### Outcomes

A total of 19 (52.8%) patients were discharged after full recovery (Figure 5). The overall case fatality rate was 41.7% (n = 15). All five patients with CCHF survived with no severe complications, whereas 48.3% (n = 15) of those with RVF died in the hospital. Two (5.6%) patients eloped from the isolation unit without formal discharge before full recovery. Nine (75%) of the 12 patients who underwent hemodialysis were discharged alive, whereas five of eight (62.5%) patients with stage 3 AKI who did not undergo dialysis died in the hospital. The overall mean duration of hospital stay was 10.4 days (SD 9). However, for the patients who died in the hospital, the mean time from admission to death was only 2.4 (SD 2.75) days, whereas the time from admission to discharge for the survivors was 17.47 (SD 6.74) days. Survivors, therefore, had a significantly longer hospital stay than non-survivors (mean difference 15.07 days, 95% confidence interval 11.57-18.57; *P* < 0.001).

**Figure 5:**
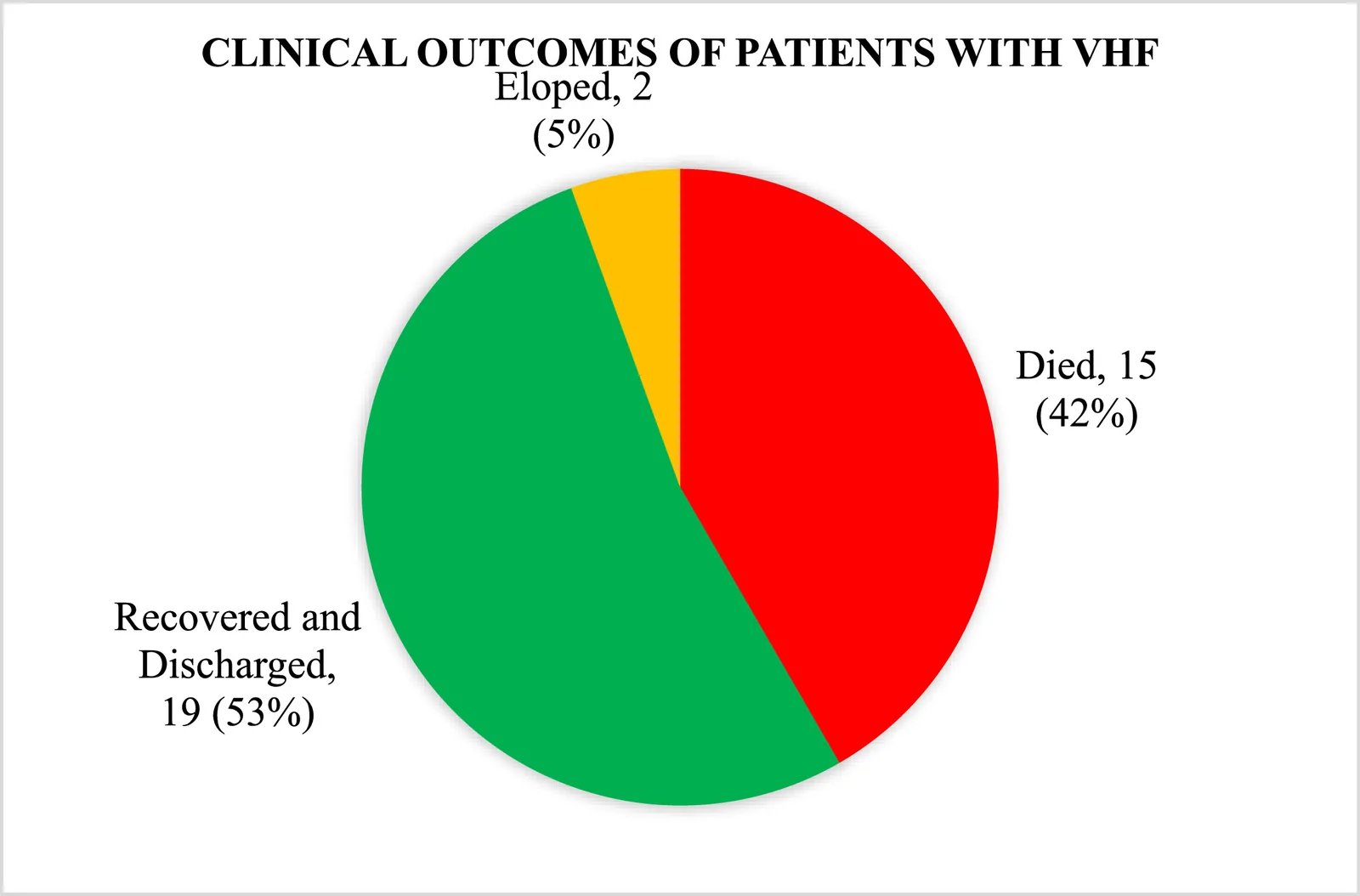
In-hospital outcomes of patients with RVF and CCHF at MRRH, southwestern Uganda. Abbreviations: RVF, Rift Valley fever; CCHF, Crimean-Congo hemorrhagic fever; MRRH, Mbarara Regional Referral Hospital.

**Figure 6:**
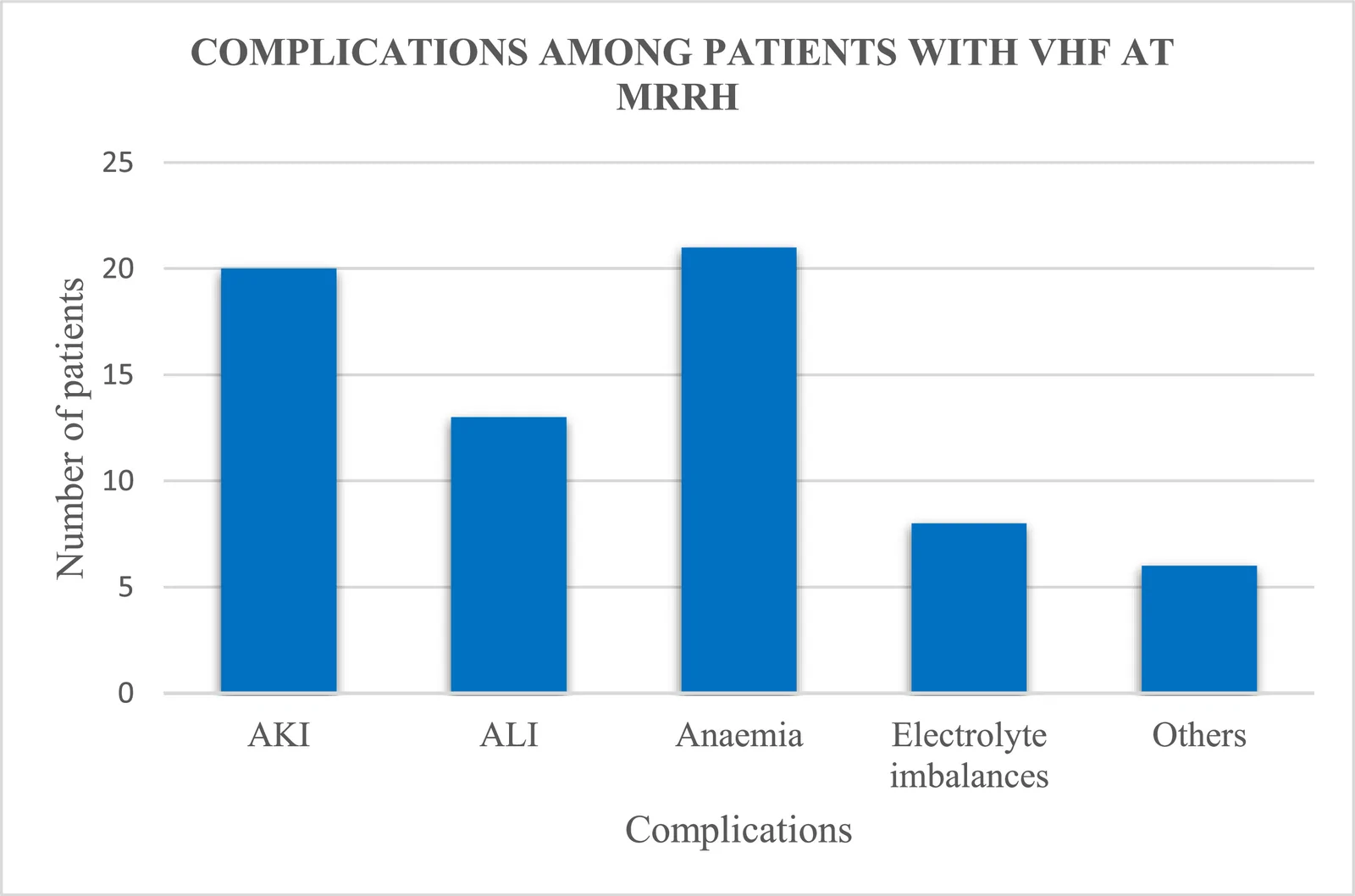
Complications among patients with RVF and CCHF at MRRH, southwestern Uganda. Abbreviations: RVF, Rift Valley fever; CCHF, Crimean-Congo hemorrhagic fever; MRRH, Mbarara Regional Referral Hospital.

## Discussion

This study’s findings demonstrate the various presentations and outcomes among patients with VHF. The male predominance (88.9%) and the occupational risks among cattle keepers and butchers underscore the zoonotic nature of RVF and CCHF, whereby occupational and behavioral exposures—more so among livestock handlers, herders, and butchers—place men at a significantly higher risk. Similar studies carried out in Kenya, Tanzania, Sudan, and Uganda reported very high male-to-female ratios of up to 3:1 [[22], [23], [24]]. In our study, two of every three patients interacted directly with animals and/or animal products, with cattle keepers, herdsmen, and butchers being the most represented occupations. The study site being in the cattle corridor further underscores the need for tailored prevention programs.

Bleeding manifestations were reported in 77.8% of the patients in this study. This rate of bleeding is higher than rates reported earlier in many studies, signifying higher rates of severe disease in southwestern Uganda. For instance, a case series describing an RVF outbreak in Sudan reported a lower rate of bleeding tendencies of 27.8% [24]. The presence of thrombocytopenia, elevated INR, and ALI, explains the multifactorial pathogenesis of bleeding and disseminated intravascular coagulation in patients with VHF, which involves endothelial damage, hepatic dysfunction, and platelet consumption [25,26]. In a recent systematic review, Anywaine et al. [16] found fever (81%), renal failure (41%), nausea (38%), jaundice (26%), hemorrhagic disease (26%), partial blindness (24%), encephalitis (21%), cough (4%), and miscarriage (54%) to be the most prevalent manifestations.

The majority of patients in this population had severe AKI, with serum creatinine levels indicating stage 3 AKI. However, only about half of these patients underwent hemodialysis due to cost constraints because dialysis is an expensive paid-for service at our facility. Notably, over half of those who required dialysis but did not receive it died. However, given the small sample size, this study was not sufficiently powered to determine causal relationships between dialysis and survival. The elevated levels of AST and ALT, hyperbilirubinemia, and coagulopathy in this population also indicate severe ALI caused by these infections, a hallmark of advanced disease. These findings are similar to those described in a systematic review by Guevara et al. [27], where 50% of the patients with VHF had AKI requiring renal replacement therapy. Furthermore, we have more recently reported similar findings of severe AKI and ALI in a case series of four patients with RVF [14].

The 41.7% mortality rate is comparable to mortality rates in other East African outbreaks [8,9], particularly for hospitalized patients with bleeding and multi-organ dysfunction. Factors such as late presentation (i.e. median duration of symptoms before hospitalization of 5 days), resource limitations, and delayed access to critical care may have contributed. Nonetheless, the survival of over half the patients (53%) and all patients with CCHF, despite these challenges, illustrates the potential benefit of structured isolation units and supportive care in the treatment of patients with VHFs. The authors also found considerable early in-hospital mortality, which was likely due to late presentation with severe forms of disease.

### Limitations

The small sample size limited the power of this study to address some objectives, such as the association between individual patient characteristics, management modalities, and outcomes. Given the very small number of CCHF cases in this study (n = 5), the authors could not objectively make a statistical comparison between survival rates for RVF and CCHF. This was also a single-center study, which limits the generalizability of the study’s findings to other patients with VHF in other parts of the world. Follow-up patients for long-term outcomes after recovery from RVF or CCHF was not performed. Furthermore, the authors did not have access to postmortem reports to ascertain the causes of death in particular cases of RVF. Finally, because not all patients had records of baseline kidney and liver function, interpretation of laboratory results was made on the assumption that the patients had no underlying kidney or liver disease. This may have resulted in the over-interpretation of elevations in biochemical markers for acute kidney and liver injury.

## Conclusion

Patients with VHFs at MRRH presented with severe manifestations of multi-organ involvement, especially hepatic and renal dysfunction. The high case fatality rate highlights the need for early detection, occupational risk mitigation, and improved supportive care infrastructure. This study’s findings also support the inclusion of VHF in the differential diagnoses of patients presenting with fever, bleeding, jaundice, or AKI in the endemic regions of East Africa.

### Recommendations

Future research should explore predictive models of severe disease and evaluate outcomes with enhanced therapeutic interventions. Further studies should also focus on describing the long-term outcomes of survivors of RVF and CCHF to guide the development of holistic care guidelines. Given the high incidence of AKI in our patient population, we recommend efforts to make renal replacement therapy for patients with severe VHF, such as dialysis, more available and affordable. Finally, the authors recommend designing tailored evidence-based treatment protocols for endemic VHFs, such as RVF and CCHF, in East Africa.

## Declarations of competing interest

The authors have no competing interests to declare.

## Funding

This research did not receive any specific grant from funding agencies in the public, commercial, or not-for-profit sectors.

## Ethical approval

This study was performed in compliance with relevant laws, regulatory frameworks, and guidelines where the research took place. This study was approved by the MUST Research Ethics Committee (approval number MUST-2024-1552).

## Availability of data and materials

The complete data sets for this study are available by the corresponding author upon reasonable request.

## CRediT authorship contribution statement

**Rose Muhindo:** Conceptualization, Formal analysis, Methodology, Project administration, Resources, Supervision, Writing – original draft, Writing – review & editing. **Ernest Ssewanyana:** Data curation, Formal analysis, Investigation, Visualization, Writing – original draft, Writing – review & editing. **Badru Kayongo:** Conceptualization, Data curation, Writing – review & editing. **Andrew Mutekanga:** Formal analysis, Methodology, Software, Writing – original draft, Writing – review & editing. **Francis Mugabi:** Investigation, Writing – review & editing. **Precious Twesigye:** Conceptualization, Data curation, Writing – original draft, Writing – review & editing. **Jeremiah Lwasa:** Writing – original draft, Writing – review & editing. **Nelson Wandera:** Conceptualization, Data curation, Investigation, Writing – original draft, Writing – review & editing. **Peter K. Olds:** Conceptualization, Supervision, Writing – original draft, Writing – review & editing. **Louise C. Ivers:** Conceptualization, Supervision, Writing – original draft, Writing – review & editing.
